# Spoken language outcome measures for treatment studies in Down syndrome: feasibility, practice effects, test-retest reliability, and construct validity of variables generated from expressive language sampling

**DOI:** 10.1186/s11689-021-09361-6

**Published:** 2021-04-08

**Authors:** Angela John Thurman, Jamie O. Edgin, Stephanie L. Sherman, Audra Sterling, Andrea McDuffie, Elizabeth Berry-Kravis, Debra Hamilton, Leonard Abbeduto

**Affiliations:** 1 MIND Institute, University of California Davis Health, 2825 50th Street, Sacramento, CA USA; 2 Department of Psychiatry and Behavioral Sciences, University of California Davis Health, Sacramento, CA USA; 3 Department of Psychology, University of Arizona, Tucson, AZ USA; 4 Department of Human Genetics, Emory University, Atlanta, GA USA; 5 Waisman Center and Department of Communication Sciences and Disorders, University of Wisconsin-Madison, Madison, WI USA; 6 Departments of Pediatrics, Neurological Sciences and Biochemistry, Rush University Medical Center, Chicago, IL USA

**Keywords:** Down syndrome, Outcome measures, Clinical trials, Treatment, Expressive language sampling, Expressive language, Psychometrics

## Abstract

**Background:**

The purpose of this study was to evaluate expressive language sampling (ELS) as a procedure for generating spoken language outcome measures for treatment research in Down syndrome (DS). We addressed (a) feasibility, (b) practice effects across two short-term administrations, (c) test-retest reliability across two short-term administrations, (d) convergent and discriminant construct validity, and (e) considered comparisons across the conversation and narration contexts.

**Method:**

Participants were 107 individuals with DS between 6 and 23 years of age who presented with intellectual disability (IQ < 70). The utility of ELS procedures designed to collect samples of spoken language in conversation and narration were evaluated separately. Variables of talkativeness, vocabulary, syntax, utterance planning, and articulation quality, derived from transcripts segmented into C-units (i.e., an independent clause and its modifiers), were considered. A 4-week interval was used to assess practice effects and test-retest reliability. Standardized direct assessments and informant report measures were collected to evaluate construct validity of the ELS variables.

**Results:**

Low rates of noncompliance were observed; youth who were under 12 years of age, had phrase-level speech or less, and had a 4-year-old developmental level or less were at particular risk for experiencing difficulty completing the ELS procedures. Minimal practice effects and strong test-retest reliability across the 4-week test-retest interval was observed. The vocabulary, syntax, and speech intelligibility variables demonstrated strong convergent and discriminant validity. Although significant correlations were found between the variables derived from both the conversation and narration contexts, some differences were noted.

**Conclusion:**

The ELS procedures considered were feasible and yielded variables with adequate psychometric properties for most individuals with DS between 6 and 23 years old. That said, studies of outcome measures appropriate for individuals with DS with more limited spoken language skills are needed. Context differences were observed in ELS variables suggest that comprehensive evaluation of expressive language is likely best obtained when utilizing both contexts.

## Background

With an estimated prevalence of 1 in 691 live births, Down syndrome (DS) is the leading genetic cause of intellectual disability (ID [1];). Heterogeneity is observed at every level of description and stage of development. Nevertheless, individuals with DS often present with some core developmental challenges, with expressive language skills constituting the most affected aspect of development. Language delays are observed in virtually all individuals with DS and the severity of language delay is often greater than those measured in other ID-associated conditions [2–5]. Moreover, because language skills play a critical role in social functioning, cognitive development, academic achievement, and daily living skills, language delays are arguably the greatest barrier to independence and meaningful community inclusion for individuals with DS. Thus, treatments leading to improvements in communication are likely to have widespread benefits, improve quality of life, and be of high priority to develop and evaluate. In fact, clinical trials of targeted treatments in DS are underway with at least 16 (completed, active, or openly recruiting) studies reported on clinicaltrials.gov utilizing language/communication as an outcome measure.

In recent years, significant advances have been made in elucidating the mechanisms underlying development across multiple levels of analysis, from cellular to behavioral, and have fueled efforts for the development of a more robust DS translational research agenda nationwide [6]. Indeed, promising results from studies considering pharmaceutical treatments in the DS Ts65Dn mouse model [7, 8] indicate that a diverse array of treatments demonstrate at least the partial rescue of learning and memory difficulties. With these positive findings, more human clinical trials are imminent. Increased efforts are also being directed toward the development and evaluation of behavioral, educational, and psychosocial interventions [9–12].

At the same time, there is a growing body of research highlighting a critical hurdle in the development and success of clinical trials; namely, the identification and availability of appropriate and meaningful outcome measures [13, 14]. This literature has largely emerged in response to the fact that preclinical successes have failed to translate into successful human trials [15, 16]. Indeed, there are limitations associated with many of the tools available for assessing cognitive changes in individuals with intellectual and developmental disabilities (IDD). For example, many of the standardized assessments available for assessing developmental changes have not been specifically validated or designed for use in IDD populations [17, 18]. As a result, many individuals with IDD score at the floor of the assessment, which precludes obtaining an accurate baseline level or assessing magnitude of change. Another limitation of standardized assessments is that they assess skills in contexts removed from daily life. Thus, performance on standardized assessments may not generalize to performance in real-world contexts or reflect changes that are meaningful to the individuals.

The lack of adequate outcome measures validated for use in individuals with IDD, including DS, presents a significant obstacle to the evaluation of treatment efficacy. There is a strong need for psychometrically sound outcome measures for language that are applicable across the range of heterogeneity demonstrated by individuals with DS in order to assess treatment efficacy. Improvements in expressive language, a particular weakness in individuals with DS, are likely to have widespread benefits, improve quality of life, and be of high priority for families. In the present study, we evaluated expressive language sampling (ELS) as a procedure for generating spoken language outcome measures for treatment research in DS.

### Expressive language sampling

ELS refers to a set of procedures used frequently in research and clinical practice to characterize developmental changes and impairments in expressive communication [19]. These types of procedures have been identified as promising options for clinical trials by multiple NIH-convened working groups charged with the task of evaluating and proposing potential outcome measures for use in IDD groups [13, 20, 21]. Indeed, the specific ELS procedures, and associated variables, we consider in the present study for use in DS have been previously reported to demonstrate promising feasibility and adequate psychometric properties in individuals with fragile X syndrome (FXS) ranging in age from 6 to 23 years [19].

In general, ELS procedures offer an attractive alternative to norm-referenced standardized assessments. In ELS, samples of participant talk are collected in structured, yet naturalistic interactions with a partner. In the present study, as in Abbeduto et al. [19], samples were collected using scripts to minimize variation in format, content, and the behavior of the examiner, thereby ensuring reasonable consistency across participants, assessment timepoints, and examiners. Once collected, samples are transcribed into electronic text files according to standard conventions, allowing analysis via computer-based algorithms to derive numerous clinical endpoints. There are multiple advantages to ELS compared to typical standardized tests of language skills. For example, ELS procedures (1) use a format more closely aligned with real-world contexts and, therefore, are more likely to generalize to activities that are functional and meaningful for the participant [22]; (2) can yield multiple dependent variables, reflecting different domains of skill, that can be examined separately or organized into composites (making the procedure flexible for clinical trials and for considering developmental changes when significant heterogeneity among participants is likely); (3) are less prone to noncompliance and floor effects as compared to standardized tests; and (4) can be collected quickly and often with minimal training of examiners, making ELS especially attractive for multi-site trials [19].

Recently, Abbeduto et al. [19] evaluated the psychometric properties of ELS variables, generated from a conversation and narration task, for individuals with FXS and comorbid ID, ranging in age from 6 to 23 years. More specifically, the authors assessed the feasibility, occurrence of practice effects and reliability across the test and retest administrations, and construct validity. Overall, evaluation of the ELS procedures in this sample of youth with FXS demonstrated compliance rates of over 85% on both procedures. Participant factors found to be associated with increased noncompliance included a lower chronological age, more severe cognitive delays, and more severe ASD symptomatology [19]. Minimal practice effects were observed on many of the variables computed from the ELS procedures, and strong test-retest reliability was observed across a 4-week interval. Strong evidence of convergent construct validity was observed for the ELS variables of lexical diversity, syntax, and unintelligibility. Psychometric properties were found to be similar across variables generated from the two ELS procedures considered (i.e., conversation and narration).

### ELS in Down syndrome

There is reason to be hopeful, given the positive findings for FXS, that ELS procedures will be a useful tool in studies of individuals with DS. However, the psychometric properties of ELS variables must be considered in individuals with DS to understand if and for whom these procedures are appropriate. Indeed, although commonalities are observed between the FXS and DS phenotypes, there are key differences that differentiate these two phenotypes (and these conditions from other IDD populations). For example, in terms of language, differences are often noted in both the severity of delay and the profile of skills across language domains (e.g., phonology, syntax, semantics, pragmatics) between individuals with DS and individuals with FXS [23]. Importantly, there are also notable phenotypic differences outside the area of language that have the potential to influence measurement outcomes. For example, notable differences are observed between the DS and FXS phenotypes in terms of medical comorbidities, the co-occurrence of challenging behaviors and psychiatric conditions, and their associated socio-emotional phenotypes [24, 25]. Finally, the considerable heterogeneity observed among individuals with DS is also likely to influence measurement outcomes and, therefore, warrants specific attention [26].

To date, multiple studies have used the ELS procedures considered in the present study to assess expressive language skills in individuals with DS. Using these procedures, limitations in expressive language skills have been documented relative to both peers with typical development (TD) of similar cognitive developmental level and youth with FXS of similar chronological age and/or nonverbal ability level [3–5, 27]. Although each of these studies has contributed to our understanding of language skills in youth with DS by using the ELS procedures considered in the present study, much remains to be understood regarding the appropriateness and utility of these procedures in DS. More specifically, further research is needed to determine specifically for individuals with DS whether the ELS procedures (1) are feasible (i.e., appropriate across the range of ability levels demonstrated by youth with DS, as reflected in low noncompliance rates and samples of adequate length); (2) are subject to limited practice effects over repeated administrations; (3) are reliable over repeated administrations (i.e., yield high agreement between repeated administrations on the same sample of participants); and (4) demonstrate evidence of the construct validity of the measures (e.g., each ELS measure should correlate with other measures of the same ability or attribute). Finally, if the variables derived from ELS are found to meet all of these criteria, it will be important to demonstrate that these variables are also sensitive to change, particularly in situations and treatments in which change is known to occur.

### The present study

In the present study, we focused on the initial results from an ongoing multi-site study of the psychometric properties of ELS-derived variables for a large cohort of US, English-speaking individuals with DS, ranging in age from 6 to 23 years. The following research questions were considered:

1. Are the ELS procedures feasible, defined in terms of participant compliance rates and amount of talk produced, for youth with DS between 6 and 23 years of age, such that the samples are adequate for the majority of youth with DS considered? In addition, we considered whether the ELS procedures were equally feasible the full range of participant ages, IQs, etc. represented in the sample.
2. Are minimal practice effects and adequate test-retest reliability observed for each of the ELS-derived variables across the two, short term interval administrations?
3. Can convergent and discriminant validity be established for each of the ELS-derived variables?
4. Are there significant differences in the ELS variable scores generated from the ELS conversation and narration tasks?

## Methods

### Participants

Participants with DS between the ages of 6 and 23 years were enrolled in this study. This study parallels the design reported by Abbeduto et al. [19], with the lower bound of this age band adopted based on expectations concerning the limited capacity of children with ID under 6 years of age to complete the battery of ELS procedures of interest and the upper bound adopted to decrease the possibility of including individuals with dementia in our DS group. All participants provided medical reports documenting trisomy 21 or translocation, with or without mosaicism. Additional inclusion criteria, based on caregiver report, were (1) willingness of both the youth participant and parent/caretaker to participate in the protocol; (2) use of speech as primary mode of communication for the youth participant, with multi-word utterances used at least occasionally; (3) English as the primary language used in the home; (4) no more than a mild hearing loss; and (5) no serious (uncorrected) visual impairment that would preclude successful performance on the testing battery. In addition, all youth participants had an IQ within the ID range (IQ ≤ 70), first determined using parent report and record review and subsequently confirmed via direct testing at the study Time 1 study visit (described below).

In addition, participants could not be actively enrolled in a randomized clinical trial for the 8 weeks prior to the initial testing visit and the period between the initial testing and retesting visit (~ 4-week interval). Moreover, all attempts were made to schedule participants to avoid a change in physician-prescribed medications designed to manage behavior (e.g., SSRIs), an open-label clinical trial medication, behavioral therapy, or educational programming (not including regular school holidays/vacations) between the initial and retest visits, as well as in the 8 weeks preceding the initial testing visit. Using this approach, no participants needed to be excluded from analyses.

Four university testing sites collectively recruited and tested 107 participants with DS (55 males, 52 females), with a mean age of 15.13 years (*SD* = 5.15, range 6.45–23.72), a mean Stanford-Binet Intelligence Scales, Fifth Edition (SB-5) Abbreviated Battery IQ of 48.73 (*SD* = 4.39, range 47–73; note: 82 participants received a score of 47, the lowest score possible), and a mean SB-5 Abbreviated Battery IQ deviation score of 33.74 (*SD* = 14.17, range − 4.98–74.89). A deviation IQ score can be used to ameliorate floor effects, thereby improving the precision of IQ measurement in individuals with ID, by applying a raw z-score transformation of the SB-5 general population norms.

### Study design

The Institutional Review Boards of all participating universities reviewed and approved study procedures. Written informed consent from the parent/guardian of all participants and youth assent were obtained prior to starting study procedures. Initial visit study measures were administered during the course of a single day for 76% of the sample; the remaining participants completed the two halves of the initial study visit on different days, with an average of 3.81 days between study half administrations (*SD* = 5.77, range 1–22). At the initial visit, participants completed the ELS procedures as well as construct validation measures and measures focused on describing the youth characteristics (e.g., IQ, ASD symptom severity). At the retest visit 4 weeks later, only the ELS procedures were administered. All participants completed the retest visit in a single day, with a mean test-retest interval of 4.24 weeks (*SD* = 0.78, range 3.00–6.00).

### Expressive language sampling

#### Procedural overview

For the present study, we analyzed expressive language samples collected in two contexts—conversation and narration—both during the initial test visit and during the retest visit. Both order of administration (i.e., conversation before narration or narration before conversation) and task version (i.e., Version A vs. Version B) were rotated across participants at the initial test visit and across test and retest visits. With regard to order of administration, 56 participants received conversation before narration and 51 participants received narration before conversation at the initial visit, with the opposite order administered at the retest visit. With regard to version, 53 participants received Version A (of conversation and narration) and 54 participants received Version B at the initial test visit, with the opposite order administered at the retest visit.

In a prior study focused on FXS, we provided considerable detail regarding these ELS procedures [19]. In review, the conversation and narration ELS procedures considered in the present project were designed to elicit spontaneous speech in naturalistic contexts, while following procedures that created reasonable standardization of the context and examiner behavior to ensure comparability across participants and occasions of measurement. Indeed, prior research has demonstrated that variation in both materials, context, and examiner influence can have dramatic effects on the expressive language produced by speakers from children to adults and for individuals with typical as well as delayed development [28]; thus, balancing standardization with naturalness is a critical component of ELS procedures.

All examiners who administered the ELS procedures in the present project completed a standardized training process, under the supervision of the lead site, to ensure fidelity of the administration procedures [19]. This process involved (1) an in-person training with a team from the lead site, (2) self-guided review of written instructional manuals and video recordings of “gold standard” administrations, and (3) self-guided practice with adult peers with TD to become comfortable with the materials and procedures. Throughout this process, questions from examiners-in-training were answered as needed. Following this review process, all examiners were required to demonstrate a fidelity administration (90% or higher) with both a young participant with TD and a participant with developmental delays according to a standardized scoring rubric. Examiners trained on the current project included undergraduate research assistants, staff, and professionals (e.g., psychologists, speech-language pathologists, etc.) familiar with youth and families with intellectual and developmental disabilities and needed to submit at most two administrations, in each group, to achieve fidelity. Manuals describing the ELS procedures for administration, training, and assessment fidelity are available at https://ctscassist.ucdmc.ucdavis.edu/ctscassist/surveys/?s=W9W99JLMNX. Administration fidelity was also assessed for a subset of actual study administrations by trained examiners (13) across the different sites, with a mean fidelity rating of 94% (range 81–100%).

All ELS administrations were digitally audio-recorded and then later transcribed and analyzed by the lead site using the Systematic Analysis of Language Transcripts (SALT [29]). All transcripts were first drafted by a primary transcriber, reviewed, and edited by a secondary transcriber, and then finalized by the primary transcriber; in total, we estimate that this process takes approximately 3 h for a conversation and 2.25 h for a narration; however, considerable variability, in terms of transcription time, is observed depending on the nature of the samples (e.g., length, amount of talk, intelligibility, etc.). Talk was segmented into Communication-units (C-units), which provides a more accurate measure of language ability than does segmentation into only utterances for speakers beyond a developmental level of 3 years [30]. A C-unit is commonly defined as an independent clause and its associated modifiers, including dependent clauses. However, it is important to note that this definition indicates the upper bound of a C-unit and that non-clausal utterances such as sentence fragments and elliptical responses also constitute C-units [see also 19]. All transcribers underwent rigorous training and, following training, were required to achieve agreement with three gold standard transcripts (1 TD sample, 1 DS sample, and 1 FXS sample) prior to transcribing study data. We estimate this training and reliability process takes 4–6 months; for example, our most recent cohort of transcribers, all of whom worked part time on the project (50%), took an average of 4.96 months (range 4.30–6) to complete.

Transcribers on the present project were blind to diagnosis, test visit, and results of other measures completed by the participant. Inter-transcriber agreement was randomly assessed for 10 transcripts (4 Narration, 6 Conversations), with at least three from each age group and two from each site. Considering the dimensions of transcription that impact the variables considered in this study, inter-transcriber agreement was observed to be 87% for utterance segmentation, 87% for identification of partly or fully unintelligible C-units, 90% for identification of C-units containing mazes, and 84% for identification of the exact lexical and morphemic content of each C-unit. In addition, inter-transcriber agreement was 76% for identification of the exact number of morphemes in each C-unit and 80% for the exact number of words in each C-unit. However, an outlier was observed in the reliability scores, without this outlier agreement was 80% for identification of the exact number of morphemes in each C-unit and 84% for the exact number of words in each C-unit. These agreement percentages are consistent with those previously reported within the literature for these tasks [19, 27, 30, 31].

#### Conversation

As described in detail by Abbeduto et al. [19], the conversation task consisted of an interview-style conversation with the examiner. Within this task, the examiner uses mainly open-ended prompts (e.g., “Tell me everything you did at school yesterday), uses broad follow-up questions and prompts (e.g., what do you like about [topic]?), and limits his/her own speech during the sample, with the goal of encouraging as much youth talk as possible across a 12-min period. In addition, the examiner follows a scripted order of topics to discuss with the youth. Sessions begin by inquiring about a topic (based on caregiver report) of interest to the youth (e.g., “I was talking with your mom and she told me that you love going on nature walks. That sounds very interesting. Tell me about that.”). After no more than 3 min (and typically less), the examiner moves on to the first topic assigned to the administration order. At least three topics, in addition to the youth’s topic of interest, are attempted within the allotted period. In addition, a minimum of one to two follow-up prompts are attempted for each topic before moving on (e.g., no response or when the topic does not engage or seem of interest to the participant). In instances in which the full topic list is exhausted prior to reaching 12 min, the examiner can introduce up to two more topics that were reported by the caregiver as being of interest to the youth. In instances in which the youth introduces additional topics, the examiner maintains the topic by using appropriate open-ended follow-up prompts (e.g., “Tell me more.”). There are two different versions of the Conversation task (A and B) for both “School-Age” and “Adult” participants, allowing alternate versions to be used in test and retest visits. Based on preliminary work, slightly different sets of topics are used between the “School-Age” and “Adult” versions (e.g., school is a useful topic for the former, but not the latter); the procedures are otherwise identical for participants of different ages.

#### Narration

As described in detail by Abbeduto et al. [19], the narration task consists of the participant telling the story from a wordless picture book. Within this task, the examiner introduces the activity and has the participant look at each page spread silently to get a sense of the story; during this phase, the examiner allows the youth ~ 10 s to review the page spread before turning the page. Once the first showing is completed, the examiner asks the participant to tell the story. The examiner controls the book and waits until the participant has finished his/her description before turning the page (i.e., turning the page 5 to 7 s after the participant has finished talking). Similar to the conversation task, examiner prompts, and responses are standardized. There is no predetermined time limit for administration of the narration task. Two books, each including 16 pages of story content from the Mercer Mayer’s “Frog” series, were used during the narration task: *Frog Goes to Dinner* (Version A) and *Frog on His Own* (Version B). Prior investigations have shown these versions to yield comparable dependent variables [27].

#### ELS variables

Five primary variables were computed for each language sample. Each variable was computed automatically by SALT or with minimal transformation of SALT-generated variables (e.g., computation of a percent).

##### Lexical diversity

This variable provides an estimate of the size of the participant’s expressive vocabulary and is computed as the number of different word roots in 50 complete and fully intelligible C-units (or the full sample of complete and fully intelligible C-units produced if less than 50 C-units were produced).

##### Syntax

This variable provides an omnibus measure of syntactic maturity and is computed as the mean number of morphemes per C-unit for complete and fully intelligible C-units.

##### Talkativeness

This variable provides an estimate of the motivation to talk and is represented by the number of C-units attempted per minute.

##### Intelligibility

This variable provides an index of difficulties in speech articulation and is computed as the proportion of the total C-units that are marked as either partly or fully intelligible by the transcriber.

##### Dysfluency

This variable provides an index of difficulties in language planning and is computed as the proportion of the total number of complete and fully intelligible C-units that include a maze or verbal dysfluency (e.g., um, uh, er, or repetition of word parts or words).

### Individual difference measures

#### Stanford-Binet Intelligence Scales, Fifth Edition

Cognitive ability was assessed using the Stanford-Binet Intelligence Scales, Fifth Edition (SB-5 [32]). This measure yields a number of different variables including abbreviated IQ, full scale IQ, nonverbal IQ, and verbal IQ scores, among others. Mean IQ for all these variables is 100 in the norming sample, with a standard deviation of 15. Due to the significant floor effects observed when utilizing the SB-5 with individuals with intellectual disabilities, we utilized the deviation IQ scores developed by Sansone et al. [33], which provides a *z-*score transformation based on the general population norms. Deviation IQ scores for Abbreviated IQ were utilized in the present analyses. In addition, performance on this measure was used to confirm the presence of ID in all participants within the present study. Data were missing for 3 participants (2 due to examiner error, 1 due to participant distress).

#### Autism Diagnostic Observation Schedule, Second Edition

The Autism Diagnostic Observation Schedule, Second Edition (ADOS-2 [34]) is a semi-structured observational context designed to observe reciprocal interaction skills in addition to the presence of repetitive behaviors. One of four ADOS-2 modules is generally administered based upon the participant’s expressive language level; due to the level of ID and associated limited independence levels observed in our sample, none of the participants met criteria for a Module 4 administration. In the present study, 6 participants received a Module 1, 47 participants received a Module 2, and 39 participants received a Module 3. The ADOS-2 was administered by research-reliable examiners. Missing data were observed for 16 participants (a research-reliable ADOS-2 examiner was not available for 13 participants, 2 participants were administered the wrong module, and 1 participant could not complete the ADOS-2 due to fatigue).

### Validation measures

#### Clinical Evaluation of Language Fundamentals, Fourth Edition, Expressive Vocabulary Subtest

The Clinical Evaluation of Language Fundamentals, Fourth Edition (CELF-4; 35) Expressive Vocabulary (EV) subtest was used to establish the convergent construct validity of the ELS variable lexical diversity. In the EV subtest, a participant’s ability to name illustrations of people, objects, and actions are considered. To minimize floor effects, raw scores were used instead of standard scores, with all participants, regardless of chronological age, starting at item 1 and continuing until the ceiling criteria were met.

#### Clinical Evaluation of Language Fundamentals, Fourth Edition, Formulated Sentences Subtest

The CELF-4 Formulated Sentences (FS) subtest [35] was used to establish the convergent validity of the ELS variable syntactic complexity. In the FS subtest, a participant’s ability to formulate a sentence about a visual stimulus using a target word or phrase was considered. To minimize floor effects, raw scores were used instead of standard scores, with all participants, regardless of chronological age, starting at item 1 and continuing until the ceiling criteria were met.

#### Goldman-Fristoe Test of Articulation, Second Edition, Sounds in Words Subtest

The Goldman-Fristoe Test of Articulation, Second Edition (GFTA-2 [36]) Sounds in Words (SiW) subtest was used to establish the convergent validity of the ELS intelligibility variable. This subtest is designed to evaluate an individual’s articulation skill when labeling single words. During administration, the examiner presents a picture stimulus for the individual to label and scores the production of target consonant and consonant cluster sounds as correct or incorrect. All SiW samples were audio-recorded and scored by the lead site. The percent of correct consonant/consonant clusters produced was used in the present analyses.

#### Vineland Adaptive Behavior Scales, Second Edition, Expressive Communication Sub-Domain

The Vineland Adaptive Behavior Scales, Second Edition (Vineland-2 [37]) Expressive Communication Sub-Domain was used to establish the convergent validity of the ELS Talkativeness variable. This sub-domain score is designed to provide a variable of a participant’s use of words and sentences to express themself verbally. The Parent/Caregiver Questionnaire version of the Vineland-2 was used. Expressive Communication subdomain raw scores were used in the present analyses. Missing data occurred for 8 participants (1 protocol was not returned, 7 protocols had missing items that impacted scoring).

#### Stanford Binet Intelligence Scales, Fifth Edition, Verbal Working Memory Subtest

The SB-5 Verbal Working Memory (VWM) subtest [32] was used in the present study to establish the convergent validity of the ELS dysfluency variable. In the VWM subtest, the ability of participants to store and manipulate verbal information and plan verbal responses is considered, with the specific processes engaged varying across items. More specifically, the lower-level items of this test require a direct imitation of phrases and sentences provided, whereas the highest-level items require the recall of the last words of previously answered questions. Z-score transformations of raw scores from this subtest were utilized in the present analyses. Missing data occurred for 8 participants (4 due to examiner error, 4 due to fatigue or noncompliance).

#### Vineland Adaptive Behavior Scales, Second Edition, Maladaptive Behavior Index

The Vineland-2 Maladaptive Behavior Index (MBI [37]) was used to establish the divergent validity for the ELS variables. This Index is designed to provide a variable of the participant’s internalizing, externalizing, and other challenging behaviors that may interfere with adaptive functioning. MBI total raw score was used in the present analyses. Missing data occurred for 9 participants (1 protocol was not returned, 8 protocols had missing items that impacted scoring).

#### Aberrant Behavior Checklist-Community

The Aberrant Behavior Checklist-Community (ABC-C [38]) was also used to establish divergent validity for the ELS variables. The ABC-C is an informant report measure designed to provide variables of maladaptive behavior across a variety of dimensions (i.e., irritability, hyperactivity, lethargy/withdrawal, stereotypy, and inappropriate speech)**.** The total raw score was used in the present analyses. Missing data occurred for 1 participant, for whom a section of the form was skipped inadvertently.

#### Analysis plan

Descriptive analyses were used to assess feasibility. Parametric analyses were conducted in order to examine the potential presence of practice effects and test-retest reliability. More specifically, (1) paired samples *t* tests were conducted to evaluate the potential presence of practice effects, and (2) test-retest reliability for the variables of interest was assessed using Pearson correlations, to indicate the association, and intraclass correlations (ICCs), to assess reproducibility (i.e., agreement) between the test and retest visits, and (3) parametric analyses were also used to assess construct and discriminant validity (i.e., zero-order bivariate correlations) as well as to compare the ELS variables across the two contexts (i.e., paired samples *t* tests).

In each of these analyses, we corrected for multiple comparisons by using Benjamini and Hochberg’s false discovery rate [39] procedures in order to maintain a familywise alpha rate of *p* ≤ 0.05; however, we also present the uncorrected *p* values to provide additional information to eventual users of these outcome measures. In applying the FDR, we corrected for familywise error rate with a family defined by participant group and sampling context; for example, in the primary analyses involving the full sample of participants, the tests for the conversational measures formed one family and those for the narrative measures a second family. Note that because some variables were not normally distributed (e.g., the unintelligibility measure was negatively skewed), we also conducted nonparametric analyses where appropriate nonparametric alternatives existed. The parametric and nonparametric analyses yielded the same pattern of findings; we present the parametric analyses in the text.

## Results

### Feasibility

To assess the extent to which the participants with DS were able to complete the conversation and narration procedures meaningfully, we utilized four operational definitions of feasibility (see Table 1). These categories of feasibility are not mutually exclusive and, therefore, there is potential overlap in the participants reflected in each of these categorical analyses. The number and percentage of participants categorized using each of these methods are presented in Table 2. Results indicate some variability in the percentage of participants for whom the task was feasible in either conversation or narration according to task, timepoint, and definition of feasibility. For example, in general, lower feasibility rates were observed for the narration task than the conversation task. In addition, lower feasibility rates were observed on retest than on the initial administration when considering the number of C-units produced in either the total sample or analysis set. Nonetheless, even when using the criterion that generated the lowest rates of feasibility, ~ 80% of the sample was able to complete the narration task and ~ 83% of the sample was able to complete the conversation task at the initial administration. Consistent with the approach utilized for the FXS sample by Abbeduto et al. [19], the analyses we report in subsequent sections of the present paper exclude only those participants who were classified as non-compliant based on examiner/transcriber report. This approach provides an evaluation of the psychometric properties of the ELS variables using the least restrictive sample possible.

**Table 1:** Operational definitions for feasibility categories

Feasibility category	Operational definition
Non-compliant Examiner/Transcriber Report	Examiners and transcribers were instructed to note behavioral observations of noncompliance, defined as explicit refusal to complete the task, no response, or repeated off-task behavior. (Note: for the narration task, transcribers also classified samples in which the participant produced a task-related C-unit on 11 pages or fewer of the 16-page spreads presented).
Total Sample Utterance Length	Samples in which the participant produced < 50 C-units in conversation or < 25 C-units in narration. (Note: this variable includes utterances that are partially or completely unintelligible, which is the sample used when calculating intelligibility).
Analysis Set Utterance Length	Samples in which the participant produced < 50 C-units in conversation or < 25 C-units in narration. (Note: this variable includes only utterances that are complete and intelligible, which is the sample used when calculating lexical diversity, syntax, and dysfluency).
Sample duration	Conversation samples that were at least 9.5 min in duration or narration samples in which the participant produced at least one task-related C-unit for each of the 16-page spreads presented.

**Table 2:** Feasibility of ELS procedures: compliance, number of C-units per sample, and completeness

ELS procedure	Test	Retest
Conversation^a^		
Non-compliant examiner/transcriber report	4/107 (3.7%)	7/105 (6.67%)
Total sample utterance length	4/107 (3.7%)	1/105 (0.95%)
Analysis set utterance length	19/107 (17.76%)	5/105 (1.90%)
Sample duration^a^	1/107 (0.9%)	1/105 (0.95%)
Narration^b^		
Non-compliant examiner/transcriber report	15/106 (14.15%)	15/105 (14.29%)
Total sample utterance length	10/106 (9.43%)	7/105 (6.67%)
Analysis set utterance length	22/106 (20.76%)	16/105 (15.24%)
Sample duration^c^	12 NC/7 partial (17.92%)	13 NC/6 partial (18.10%)

Cell values indicate number (and percentages) of participants

^a^Two Time 2 Conversations missing due to youth not returning for visit

^b^One Time 1 Narration missing due to examiner error, two Time 2 Narrations missing due to youth not returning for visit

^c^Defined in terms of time for conversation and coverage of pages for narration

In total, we observed that 9 participants were categorized as non-compliant for the conversation task and 25 for the narration task (note: 1 (for conversation) and 5 (for narration) were non-compliant at both time points). These 34 non-compliant participants were not included in any of the remaining study analyses. We reviewed the characteristics of these participants, specifically focusing on CA, SB-5 ABIQ change sensitive score/age-equivalent score, language level (using the ADOS-2 module as a proxy), and ASD classification. We found that all participants deemed non-compliant for the conversation task, and all but 4 participants on the narration task, were in the youngest age bracket tested (i.e., 6–11 years old). Considering the participants in the present study who were under 12 years of age, ~ 24% and ~ 55% were non-compliant on the conversation and narration tasks, respectively. In addition, we found that all participants who were non-compliant for either the conversation or narration task earned an SB-5 ABIQ change sensitive score of 468 or less, which reflects an ABIQ age-equivalent score of less than or equal to 4 years, 9 months. There were 63% of participants in the overall study who earned an SB-5 ABIQ score within this range (with data missing for 2 participants), leading to an estimate of ~ 14% and ~ 40% of youth in this developmental range were non-compliant for the conversation and narration tasks, respectively. When considering the ADOS-2 module as a proxy for language level, all but 1 non-compliant participants received an ADOS-2 Module 1 or 2, indicating a language level of phrase speech or less. When considering only the participants who received these modules in the present study (data missing for 12 participants), we found that 16% and 42% were non-compliant on the conversation and narration tasks, respectively. Unlike the other participant characteristics, ASD classification on the ADOS-2 did not appear to be a key contributor to non-compliance, with the proportion of youth with noncompliant samples classified in the ASD range (conversation: 22%, narration: 36%) being similar to the ASD classification rate observed for the whole sample (36%). Finally, considering only the participants who met all three of the primary factors that appear to relate to likely non-compliance (i.e., age less than 12 years, ABIQ age equivalent score < 4.75, and ADOS-2 module < 3), we found that 38% and 75% were non-compliant on the conversation and narration tasks, respectively. In contrast, considering only those participants who did not meet all three of these criteria, we found that none were non-compliant on conversation and 5% were non-compliant on narration.

### Practice effects and test-retest reliability

Next, we considered the presence of practice effects between the initial test visit and the retest visit. For each task, only those participants who were compliant on both administrations were included in these analyses. As seen in Table 3, when considering performance on the conversation task, results of paired samples *t* tests indicated no significant difference between test and retest scores for the syntax, talkativeness, unintelligibility, and dysfluency variables. This same pattern of findings was also observed in the narration task (see Table 3). For all variables, Cohen’s *d* was observed to be less than .17. When considering the lexical diversity variable, a significant increase in scores was observed across the two time points for the conversation task, but not the narration task. This difference, however, did not remain significant after the application of the FDR. Thus, relatively little change was observed in the measures between the first and second administrations, for either conversation or narration.

**Table 3:** Practice effects on repeated administrations over a 4-week interval

	Conversation (*n* = 96)					Narration (*n* = 80)				
	Effect size	Visit 1		Retest		Effect size	Visit 1		Retest	
	Cohen’s *d*	*M*	SD	*M*	SD	Cohen’s *d*	*M*	SD	*M*	SD
Lexical diversity	− .13	74.14*	33.60	78.54*	33.50	− .09	69.68	33.01	72.80	33.34
Syntax	− .10	3.39	1.56	3.55	1.64	− .06	4.82	2.01	4.94	2.18
Talkativeness	− .01	13.00	4.10	13.05	3.54	− .17	9.51	3.57	10.12	3.57
Unintelligibility	.02	0.26	0.19	0.25	0.19	− .04	0.20	0.19	0.21	0.19
Dysfluency	− .08	0.25	0.15	0.26	0.15	− .10	0.25	0.15	0.26	0.16

* prior to FDR correction, p = .018; not significant after FDR correction

We also considered the correlation, using simple bivariate correlations, and agreement, using ICCs, between the ELS variables at the initial test visit and the retest visit for both the conversation and narration tasks. As seen in Table 4, strong bivariate correlations and strong ICCs were observed between the initial and retest visits in the participants’ scores on each of the measures in both tasks. All bivariate correlations and ICCs were significant, even after applying the FDR procedure.

**Table 4:** Test-retest reliability over a 4-week interval: bivariate correlations and intraclass correlations

	Conversation (*n* = 96)		Narration (*n* = 80)	
	*r*	ICC	r	ICC
Lexical Diversity	.86***	.92***	.88***	.93***
Syntax	.87***	.93***	.90***	.95***
Talkativeness	.75***	.86***	.65***	.79***
Unintelligibility	.82***	.90***	.72***	.87***
Dysfluency	.77***	.87***	.76***	.84***

All correlations remain significant after FDR correction

### Convergent and discriminant construct validity

To assess construct validity, we computed the zero-order bivariate correlations between the variables computed from the initial test visit and the study validation measures. To facilitate comparison, only those participants who were compliant on both the initial and retest administrations for the task considered were included in analyses. Correlation results are presented in Tables 5 and 6 for conversation and narration, respectively. Zero-order bivariate correlations were considered; nonparametric correlations were examined for both dysfluency and unintelligibility and yielded the same pattern of findings. The diagonals in both Table 5 and Table 6 (boldface type) contain the correlations between the ELS variables and the primary external validation measures administered to establish convergent validity. Strong convergent validity was observed for the lexical diversity, syntactic, and unintelligibility measures for both conversation and narration. All of these associations remained significant after applying the FDR correction. In contrast, with nonsignificant correlations at essentially zero, convergent validity was not established for the talkativeness and dysfluency variables. With regard to dysfluency, the zero-order bivariate correlation (uncorrected) with SB-5 VWM was significant for conversation (*r* = .287, *p* = .006), but not narration (*r* = .212, *p* = .071); however, there is considerable evidence that this variable is positively correlated with syntactic complexity. Thus, we controlled for syntactic complexity when considering the association between SB-5 VWM and dysfluency; this partial correlation is reported in Tables 5 and 6.

**Table 5:** Construct validity: Conversation (n = 96)

Measures	CELF EV	CELF FS	Vineland EC	GFTA SiW	SB5 VWM
Lexical diversity	.64***	.60***	.44***	.69***	.63***
Syntax	.58***	.56***	.40***	.61***	.64***
Talkativeness	.22*	.27**	− **.13**	.05	.10
Unintelligibility	− .62***	− .50***	− .32**	− .56***	− .56***
Dysfluency	.32**	.23*	.13	.42***	− **.11**

Note that all values are bivariate zero-order correlations (uncorrected) except for that between dysfluency and the SB5 VWM score, which is a partial correlation controlling for syntax (MLU). All significant correlations remain after FDR correction

***p < .001, **p < .01, *p < .05

**Table 6:** Construct validity: narration (*n* = 80)

Measures	CELF EV	CELF FS	Vineland EC	GFTA SiW	SB5 VWM
Lexical diversity	.70***	.62***	.39**	.63***	.69***
Syntax	.70***	.62***	.42**	.65***	.71***
Talkativeness	.10	.08	− **.07**	− .05	.04
Unintelligibility	− .51***	− .37**	− .20	− .51***	− .51***
Dysfluency	.29**	.20	.11	.27*	**.18**

Note that all values are bivariate zero-order correlations (uncorrected) except for that between dysfluency and the SB5 VWM score, which is a partial correlation controlling for syntax (MLU). All significant correlations remain after FDR correction

***p < .001, **p < .01, *p < .05

For the sake of completeness, we also present the off-diagonal correlations in Tables 5 and 6. The off-diagonal cells in Tables 5 and 6 demonstrate that the ELS variables, especially lexical diversity, syntax, and unintelligibility, correlated significantly with most of the standardized tests used to establish construct validity generally. This pattern is to be expected given the high intercorrelations observed between the ELS variables (see Table 7) and the fact that the various dimensions of language largely develop in synchrony and build on each other in both typical and atypical development.

**Table 7:** Intercorrelations among ELS measures as a function of task

	Syntax		Talkativeness		Unintelligibility		Dysfluency	
	Conversation	Narration	Conversation	Narration	Conversation	Narration	Conversation	Narration
Lexical diversity	.93***	84***	.19	.21	− .61***	− .59***	.54***	.37**
Syntax			.17	− .09	− .57***	− .56***	.53***	.47***
Talkativeness					− .01	.09	− .16	− .04
Unintelligibility							− .37***	− .30**

***p < .001, **p < .01

Discriminant validity of the ELS variables was also considered. More specifically, we considered whether the ELS variables were correlated with constructs that theoretically should demonstrate no association to these measures or at best associations that are smaller in magnitude, such as the presence of challenging behaviors. As seen in Table 8, with the exception of a significant association between the dysfluency in conversation and ABC total raw score, none of the associations were significant.

**Table 8:** Discriminant validity: correlations between ELS-variables and measures of challenging behaviors

Measures	Lexical diversity	Syntax	Talkativeness	Unintelligibility	Dysfluency
	Conversation				
ABC total raw score	− .14	− .13	− .02	.21	− .26*^a^
Vineland MBI total raw score	− .19	− .18	− .17	.20	− .05
	Narration				
ABC total raw score	− .03	− .06	.10	.10	− .13
Vineland MBI total raw score	− .08	− .02	− .05	− .08	.22

*p < .05

^a^Association does not remain significant after FDR correction

### Context comparisons

Finally, we compared performance on the ELS variables between the conversation and narration tasks, using only those participants who were compliant for both administration (i.e., test and retest) of the conversation and narration tasks. No significant differences were observed across the two contexts for the variables Intelligibility (Initial Test Visit: *t*(77) = 1.21, *p* = .23, *d* = .11; Retest Visit: *t*(77) = 1.46, *p* = .15, *d* = .11), or Dysfluency (Initial Test Visit: *t*(77) = 1.16, *p* = .25, *d* = .25; Retest Visit: *t*(77) = 0.93, *p* = .35, *d* = .08). At both test visits, results indicated that lexical diversity (Initial Test Visit: *t*(77) = 4.09, *p* < .001, *d* = .32; Retest Visit: *t*(77) = 4.22, *p* < .001, *d* = .31), and talkativeness scores (Initial Test Visit: *t*(77) = 9.85, *p* < .001, *d* = 1.06; Retest Visit: *t*(77) = 7.92, *p* < .001, *d* = .93) were higher in conversation than in narration and that syntax scores were higher in narration than in conversation (Initial Test Visit: *t*(77) = − 8.62, *p* < .001, *d* = − .62; Retest Visit: *t*(77) = − 8.32, *p* < .001, *d* = − .56). Nonetheless, significant associations were observed for each ELS variable across the two tasks at both the initial test visit and at the retest visit (see Table 9).

**Table 9:** Correlations between corresponding ELS-variables across the conversation and narration tasks

	Test visit	Retest visit
Lexical diversity	.76***	.79***
Syntax	.80***	.82***
Talkativeness	.55***	.47***
Unintelligibility	.66***	.76***
Dysfluency	.64***	.69***

***p < .001

## Discussion

Expressive language skills play a critical role in supporting positive outcomes and are arguably the greatest barrier to independent and meaningful inclusion for individuals with DS. Thus, treatments that target improving expressive language skills in individuals with DS are likely to have widespread benefits, improve quality of life, and be of high priority to develop and evaluate. However, no language measures have been validated for use with individuals with DS, presenting a significant barrier to treatment research. The goal of the present study was to evaluate the psychometric properties of five variables derived from two ELS contexts—narration and conversation—that have been recently validated for use in FXS [19], focusing in particular on (1) feasibility, (2) practice effects, (3) construct and discriminant validity, and (4) performance across the two contexts. Because the ELS interactive contexts are more closely aligned with performance in real-world contexts, performance is more likely to be generalizable to activities that are functional and meaningful for the individual. Moreover, the variables derived from these samples collectively provide an assessment of a diverse range of expressive language skills. Thus, if shown to meet psychometric standards for use as an outcome measure, ELS variables have several potential advantages compared to existing norm-referenced standardized assessments [40].

### Feasibility

We found that the vast majority of the 107 participants with DS, ages 6- to 23-years, were compliant with and meaningfully engaged by the conversation and narration tasks. At any single time-point, noncompliance rates were at most 6.5% for conversation and 14% for narration. In addition, other variables of feasibility were considered including total sample length, analysis sample length, and duration. Similar to the patterns observed for rates of noncompliance, we found that across variables the narration procedure was more challenging for participants, but that the vast majority of participants were compliant and meaningfully engaged even in narration. Overall, these data are similar to findings reported in a prior study considering the utility of ELS procedures for use in youth with FXS [19].

In addition, we expanded upon the findings of Abbeduto et al. [19] by conducting a closer examination of the youth characteristics associated with noncompliance. We found that the participants with DS in the youngest age bracket considered (i.e., 6–11 years old), with lower performance on the SB-5 Abbreviated Battery IQ (i.e., change sensitive score ≤ 468/age-equivalent score ≤ 4 years, 9 months), and more limited language skills (i.e., youth who have phrase-level speech or less) were at most risk for noncompliance. When we consider only the participants who met all three of these criteria, we found that the noncompliance rate for conversation was 37.55% and for narration was 75%. In contrast, for youth with DS who did not meet all three of these criteria, the noncompliance rate for conversation was 0% for conversation and 5.1% for narration. Thus, these criteria offer some clinical guidance as to the risk of noncompliance when administering ELS to youth with DS. Additionally, we found that noncompliance was not related to ASD diagnostic status on the ADOS-2 and thus, at least for DS, there does not appear to be a need to consider autism status as an inclusion criterion in treatment studies using ELS as an outcome measure—a finding that is different than reported for same-aged individuals with FXS [19].

### Practice effects and test-retest reliability

Test-retest stability is a critical aspect of measure selection because it impacts the ability to detect change amid other sources of variability. Results from the present study demonstrate minimal to no practice effects for the lexical diversity, syntax, talkativeness, unintelligibility, and dysfluency variables derived from either of the ELS contexts considered. With regard to test-retest reliability, results indicated that all ELS variables derived from both the conversation and narration samples, across the 4-week interval between test and retest visits, were highly correlated and highly reproducible. Although more research is needed to understand test-retest reliability beyond the 4-week interval considered in the present study, these data provide a promising indication that youth with DS maintain their rank order of scores and with the same absolute magnitude of difference. This is a critical feature to consider when selecting outcome measures for treatment studies of youth with DS.

### Convergent and discriminant construct validity

Another critical aspect of measurement selection is the establishment of construct validity. To be an effective outcome measure, it must be clear that the identified variables represent the skill that is intended. Generally speaking, this is accomplished by demonstrating that the variables being evaluated are significantly correlated with other variables designed to measure the same construct (i.e., convergent validity) and not associated, or associated to a lesser extent, with variables designed to measure constructs that are dissimilar (i.e., discriminant validity). For both conversation and narration, there was convergent validity for lexical diversity, syntax, and unintelligibility. Moreover, it is important to recognize that various dimensions of language largely develop in synchrony and build on each other in both typical and atypical development. For example, consider the high correlations observed among the ELS variables (e.g., lexical diversity, syntax, unintelligibility, and dysfluency). Consistent with this pattern, we found that the ELS variables, particularly lexical diversity, syntax, and unintelligibility, correlated significantly with most of the standardized tests considered in the present study regardless of the specific aspect of language purported to be measured by the standardized tests.

In contrast, convergent validity was not supported for the ELS variables talkativeness and dysfluency. This same pattern of findings was observed in analyses considering the construct validity of the ELS variables in youth with FXS [19]. As was discussed by Abbeduto et al., it remains unclear whether the lack of construct validity observed for these variables reflects limitations in the ELS variables themselves or limitations in the validation measures selected. For example, it is possible that a measure of executive functioning would have been better to consider for validation of ELS dysfluency [41]. It is also possible that different operationalizations of the constructs of dysfluency and talkativeness might be useful. For example, dysfluency could be defined as including filled pauses and stalls (e.g., composite of repetitions and filled pauses) but not revisions and repetition [42], whereas talkativeness might be defined as rate of pragmatically appropriate C-units rather than simply the rate of C-units. Thus, a changes in the operationalization of these ELS constructs may be warranted, and this may be particularly needed for the variable of talkativeness, as it showed the least stable across the test and retest administrations. Nevertheless, both talkativeness and dysfluency were correlated with at least some of the standardized tests administered, suggesting some utility as indicators of change in language ability. In any event, more research is needed before these variables can be recommended for use in treatment studies, at least when derived from these contexts. Moreover, it is possible, with future research, the utility of these variables could be established for individuals with DS of different ages or ability levels than examined in the present study. For example, in less mature language users, there is evidence that talkativeness can be validated [43] and considered an index of communicative growth in this developmental period [44]. More generally, it is important to recognize that participant characteristics can exert profound influences on the psychometric adequacy of any outcome measure.

We also considered discriminant validity for the five ELS variables. We found that the ELS variables generally correlated with validation measures selected from standardized assessments, even when they represented different language constructs. A similar pattern was observed for the participants with FXS by Abbeduto et al. [19]. This is to be expected due to the high intercorrelations between the different ELS variables, and different aspects of language performance more generally. We, therefore, also expanded on the approach of Abbeduto et al. [19] and considered the correlations between the ELS variables and standardized variables of challenging behavior, which theoretically should demonstrate associations that are smaller in magnitude [45]. With the exception of dysfluency, none of the ELS variables was significantly associated with the variables of challenging behavior, with notable decreases in the strength of the associations. Thus, across both contexts, lexical diversity, syntax, and unintelligibility were shown to demonstrate both convergent and discriminant validity, providing evidence that these variables indeed reflect the intended targeted abilities in youth with DS.

### Context comparisons

We compared performance on the ELS variables between the conversation and narration tasks. Results indicated no significant differences across the two contexts in intelligibility and dysfluency scores. In contrast, at both assessment points, we found that lexical diversity and talkativeness scores were significantly higher in conversation than in narration, whereas syntax scores were significantly higher in narration than in conversation. Nonetheless, each ELS variable was significantly associated across the two contexts. These findings are consistent with previous studies considering context difference in ELS variables. In particular, previous studies have shown that syntax scores elicited in narration tasks are higher than those elicited in conversation for youth with DS as well as in other populations with ID [30, 46–48]. It is posited that the opportunities provided by narrative tasks to describe the characters in relation to one another and characterize event sequences increase the likelihood of eliciting multi-clause constructions [49]. In addition, results from previous studies have also shown talkativeness scores to be higher in conversation than in narration [19, 27]. Finally, in the present study, lexical diversity scores were higher in conversation than in narration, and dysfluency scores were comparable across the two contexts. Findings from previous studies considering context differences in these scores have yielded variable findings [27, 30]. Thus, these patterns across contexts may be more variable and subject to influence from other factors (e.g., developmental level, age, diagnostic group). Nonetheless, findings from the present study indicate that particular variables may be better represented by a particular context, although significant associations in the ELS variables were identified across both contexts. In sum, comprehensive evaluation of expressive language is likely best obtained when utilizing both contexts. In addition, the nature of a treatment, and its expected effect, may impact ELS context and variable selection. For example, the narration task would be well-suited to assess change associated with a treatment approach targeting grammatical skills, whereas the conversation task would be well-suited to assess change in a treatment approach targeting lexical diversity.

### Logistical considerations

Decisions about the use of an outcome measure often must be based not only on the basis of scientific considerations but also on practical matters such as costs and resources involved. As indicated previously, the procedures for the administration, transcription, analysis, and training of staff have all been manualized for ELS, which is an important requirement for use in multi-site treatment studies. Moreover, rather than minimal training time or previous experience is required to learn to administer the ELS procedures at acceptable levels of fidelity, which also makes them attractive for use in the multi-site treatment studies. In contrast, the effort devoted to transcription of language samples and the training of transcribers could be a barrier for wide-scale adoption in treatment studies. One approach to addressing this challenge is to establish a single site for transcription rather than having each site in a multi-site study responsible for their own transcription, and this has been the approach used in several ongoing clinical trials. In the long run, however, computer-driven automated transcription is a possibility as speech recognition programs become more sophisticated and able to process speech from speakers of a range of ability levels and characteristics speech patterns [50].

### Limitations

This study has some important limitations that are worth noting. To begin, the ELS procedures considered in the present study represent two of many different approaches which vary in terms of prompts, materials available, and content discussed. As such, the present results do not necessarily extend to the other ELS procedures available outside our study protocols; each of these procedures would need to be evaluated before determining their utility in youth with DS. In addition, the present findings were obtained with a group of participants meeting a specific set of eligibility criteria. In particular, all participants were reported by caregivers to produce at least occasional multi-word utterances, were primarily English speakers, and had no more than a mild hearing loss. The findings regarding noncompliance rates suggest the current procedures may be too challenging for youth with more limited language skills than those included in the present study; thus, the development of tools for use in individuals with DS who have limited spoken language skills are a critical area of study. With regard to hearing status, more research is needed to understand the relations between hearing status in DS and performance on the ELS procedures. In addition, it is vital that future studies consider the utility of the ELS procedures in populations who are not primarily English speakers. This limitation creates a significant barrier to participation in treatment studies. In addition, it is likely that the ELS procedures considered in the present study can be translated and used effectively in other languages. Finally, it is important to note that we did not address the potential presence of interactions between youth characteristics (e.g., age, developmental level) and the variables generated from each task. In addition, we did not address whether or not the ELS variables were sensitive to changes in language skills. These are both critical next steps. As part of the larger project from which these data were collected, we intend to create subgroups of participants (defined by age and developmental level) and explore the psychometric properties of the ELS variables as a function of these subgroups and to compare the magnitude of change observed in the ELS variables to changes observed in the validation measures between the initial visit and a final test visit that occurred 2 years later. In addition, development of a study in which the delivery of an efficacious language intervention could be evaluated against performance on the ELS variables will facilitate our understanding of the extent to which the ELS variables are sensitive to change.

## Conclusions

The present study demonstrates the appropriateness of three ELS variables (i.e., lexical diversity, syntax, and intelligibility), derived from both conversation and narration contexts, as feasible for use in youth with DS between the ages of 6 and 23 years. Overall, the present validation results provide an important step toward providing a direct assessment of language performance that uses a format closely aligned with real-world contexts and still includes a standardization designed to increase consistency and minimize examiner influence on youth language produced. In general, the variables considered demonstrated minimal practice effects and, with the exception of the talkativeness variable, strong test-retest reliability. In addition, the vocabulary, syntax, and speech intelligibility variables were observed to demonstrate strong convergent and discriminant validity. Finally, although the variables derived from both the conversation and narration contexts were significantly associated with one another, some context differences were observed in scores, suggesting that comprehensive evaluation of expressive language is likely best obtained when utilizing both contexts. A critical question yet to be addressed is the extent to which the ELS variables demonstrate sensitivity to change. This question will be addressed using the collection of longitudinal data from study participants to explore natural developmental changes within each variable relative to changed observed on the standardized measures used in the present study to assess variable validation. Youth demonstrating a combination of a chronological age under 12 years, phrase-level speech or less, and a 4-year-old developmental level or less are more likely to have difficulty completing the ELS procedures. Thus, studies of outcome measures appropriate for individuals with DS with more limited spoken language skills are needed. Finally, overall, the findings from the present study are similar to prior findings considering the utility of ELS procedures for use in youth with FXS [19] and suggest that the ELS procedures may be a promising tool for use in other populations with ID.

## Data Availability

The datasets used and/or analyzed for the present paper can be made available upon a reasonable request to the corresponding author.

## Abbreviations

ABC-C: Aberrant Behavior Checklist-Community
ADOS-2: Autism Diagnostic Observation Schedule, Second Edition
CELF-4: Clinical Evaluation of Language Fundamentals, Fourth Edition
C-units: Communication units
DS: Down syndrome
ELS: Expressive language sampling
EV: Expressive vocabulary
FDR: False discovery rate
FS: Formulated sentences
FXS: Fragile X syndrome
GFTA-3: Goldman-Fristoe Test of Articulation, Second Edition
ID: Intellectual disability
IDD: Intellectual and developmental disabilities
MBI: Maladaptive Behavior Index
SALT: Systematic Analysis of Language Transcripts
SB-5: Stanford-Binet Intelligence Scales, Fifth Edition
SiW: Sounds in words
TD: Typically developing
Vineland-2: Vineland Adaptive Behavior Scales, Second Edition
